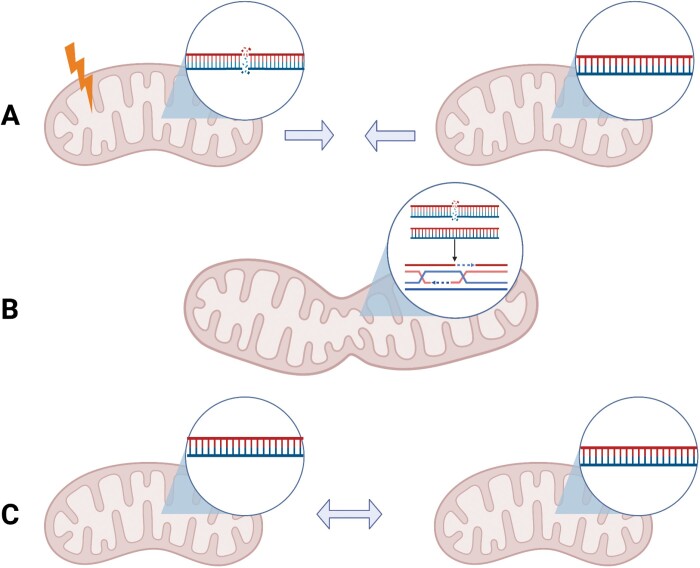# Social networks in the single cell

**DOI:** 10.1093/jxb/erac284

**Published:** 2022-09-12

**Authors:** Moira Rodriguez, Ana Martinez-Hottovy, Alan C Christensen

**Affiliations:** School of Biological Sciences, University of Nebraska-Lincoln, Lincoln, NE, USA; School of Biological Sciences, University of Nebraska-Lincoln, Lincoln, NE, USA; School of Biological Sciences, University of Nebraska-Lincoln, Lincoln, NE, USA

**Keywords:** DNA damage, DNA repair, mitochondria, mitochondrial fusion

## Abstract

This article comments on:

**Chustecki JM, Etherington RD, Gibbs DJ, Johnston IG.** 2022. Altered collective mitochondrial dynamics in the Arabidopsis *msh1* mutant compromising organelle DNA maintenance. Journal of Experimental Botany **73,**5428–5439.

**Plant mitochondrial DNA (mtDNA) can become damaged in many ways. A major repair mechanism is homologous recombination, which requires an undamaged DNA template. Presumably, this template comes from a different mitochondrion in the same cell. Plant mitochondria undergo fission and fusion to form transient networks which could allow the exchange of genetic information. To test this hypothesis, Chustecki *et al.* (2022) used *msh1* mutants with defective DNA repair, and showed that mitochondrial interactions increased, revealing a link between the physical and genetic behavior of mitochondria.**

The genomes of mitochondria (mtDNA) evolved from an encounter more than a billion years ago between an α-proteobacterium and an archaeon (Gray, 2012; Ladoukakis and Zouros, 2017). As this endosymbiotic relationship evolved, many of the mitochondrial genes were relocated to the nuclear genome or lost completely. Plant mitochondrial genomes are generally large, including abundant non-coding sequences and repeats (Ward *et al.*, 1981). Paradoxically, plant mitochondrial genomes have a very low gene mutation rate and a high rearrangement rate (Palmer and Herbon, 1988). This requires a particularly accurate mechanism for repairing mismatches and base damage.

Repair of mtDNA damage is important in both animals and plants, particularly in cell lineages that will be inherited. In animals, the female germline can sequester a subset of metabolically quiescent mitochondria, which decrease damage from reactive oxygen species (ROS), and enable the transmission of intact mtDNA to progeny (de Paula *et al.*, 2013). Plants use a different strategy. When mtDNA is damaged in plants, it is hypothesized that the damaged sequence is converted into a double-strand break (DSB), which is followed by homology-based repair using a template (Christensen, 2014) (Box 1). This ensures that complete and undamaged mitochondrial genomes will be inherited. However, the source of the DNA template for recombinational repair is still not clear. Because the copy number of mtDNA is lower than the number of mitochondria in plant leaf cells (Preuten *et al.*, 2010; Arimura, 2018), mitochondria with damaged DNA may need to fuse with other mitochondria to obtain an intact template for repair. Another possibility would be for plant mitochondria to abandon damaged DNA and let it be degraded. This occurs in some plant cells (Bendich, 2013).

In plants, MSH1, a MutS homolog, is a protein that plays a role in organellar genome maintenance, including homologous recombination (Abdelnoor *et al.*, 2003), and in *msh1* mutants the mitochondrial mutations accumulate (Wu *et al.*, 2020). By analyzing mitochondrial dynamics in the *msh1* mutant and comparing it with the wild type, Chustecki *et al.* (2022) aimed to answer the question of whether mitochondria interact differently when they have compromised mtDNA integrity. Because mitochondria in *msh1* mutants accumulate damage, the authors hypothesized that they would frequently interact with each other, possibly to acquire an intact template for homology-based DNA repair. Using fluorescently labeled mitochondria, single-cell time-lapse microscopy, and mitochondrial network analysis (Chustecki *et al.*, 2021), the authors tested their hypothesis and observed that the *msh1* mutant formed more connections within the inter-mitochondrial ‘social network’. The higher frequency of mitochondrial interactions supports increased fusion and sharing of contents when mtDNA damage accumulates, suggesting a mechanism for DNA template-directed repair.

After demonstrating the altered physical behaviors of mitochondria in *msh1* mutants, Chustecki *et al.* (2022) also compared mitochondrial behavior in *msh1* and *friendly* mutants. The FRIENDLY protein mediates inter-mitochondrial association, and *friendly* mutants exhibit a reduction in mitochondrial fusion and increased mitochondrial clustering (El Zawily *et al.*, 2014). In both *msh1* and *friendly* mutants, similar physical and social mitochondrial effects were observed, such as a smaller distance between mitochondria and increased co-localization. This supports the hypothesis that disturbances in the physical and genetic elements of plant mitochondria alter their interactions (Johnston, 2019).

While Chustecki *et al.* (2022) have answered the question of whether mitochondria physically interact within a cell in response to mtDNA damage, questions remain, and new ones arise. Although they showed that mitochondrial social interactions increase with genetic perturbations, presumably to share contents including DNA repair templates, it still needs to be shown directly that interacting mitochondria are exchanging and replicating DNA. Alongside this, it is not currently known how plant mitochondrial genomes detect genomic damage and trigger fusion with other mitochondria within the same cell. Mitochondria with DNA damage must signal to other mitochondria for fusion, but what is the nature of the signal and its reception and response? Can any mitochondrion respond to the request for a DNA template, or only a subset? Ovules must contain complete and intact copies of the mitochondrial genome—how is this accomplished, and are there checkpoints to signal the nucleus that the mitochondria are ready to be transmitted to progeny? The findings of Chustecki *et al.* (2022) open the door to further understanding of how plants accurately transmit mitochondrial DNA to their progeny.

**Box 1. Mitochondrial fusion may enable homologous DNA repair.**

(A) DNA in a mitochondrion is damaged. (B) The mitochondrion with damaged DNA fuses with another mitochondrion in the same cell and performs homologous repair with an intact template. (C) Mitochondrial fission, resulting in both mitochondria possessing the undamaged DNA copy (created with BioRender.com).